# A Pork Industry in the Backyard: An Analysis of *Toxoplasma gondii* Infection in Serbia’s Pigs

**DOI:** 10.3390/microorganisms11071857

**Published:** 2023-07-23

**Authors:** Aleksandra Uzelac, Nikola Betić, Nedjeljko Karabasil, Vladimir Ćirković, Olgica Djurković-Djaković, Ivana Klun

**Affiliations:** 1 Centre of Excellence for Food- and Vector-Borne Zoonoses, Institute for Medical Research, University of Belgrade, 11129 Belgrade, Serbia; aleksandra.uzelac@imi.bg.ac.rs (A.U.); vladimir.cirkovic@imi.bg.ac.rs (V.Ć.); olgicadj@imi.bg.ac.rs (O.D.-D.); 2 Institute of Meat Hygiene and Technology, 11000 Belgrade, Serbia; nikola.betic@inmes.rs; 3 Department of Food Hygiene and Technology, Faculty of Veterinary Medicine, University of Belgrade, 11000 Belgrade, Serbia; nedja@vet.bg.ac.rs

**Keywords:** *Toxoplasma gondii*, backyard pigs, prevalence, public health, biosecurity, oocysts

## Abstract

As pork is an important source for *Toxoplasma gondii* infection, we have analyzed *T. gondii* genotypes and toxoplasmosis prevalence in pigs in Serbia in the context of production statistics and economics to assess the specific risk to public health. Genotyping was performed using MnPCR-RFLP; *T. gondii*-specific IgG antibodies were detected using a modified agglutination test (MAT); and statistical data were extracted from official records and provided by government authorities. The results indicate that, from 2006 to 2021, the median number of annually slaughtered pigs was 5.6 million, yet only 36.1% were processed by abattoirs. The remainder were “backyard pigs” slaughtered on family farms and homesteads. Toxoplasmosis seroprevalence in market-weight (MW) pigs prior to 2006 was 15.2%, and was 15.1% in 2019. The seroprevalence in owned city cats, likely infected by livestock meat, was 33.2%. ToxoDB#1 was identified in pig tissues. The results indicate that backyard pigs are the backbone of the industry and provide as much as 60% of the pork in Serbia. The seroprevalence in pigs and city cats shows that farms are reservoirs for the parasite. Thus, innovative means of reducing *T. gondii* infection designed with backyard farmers in mind are needed to reduce the risk to public health.

## 1. Introduction

Pork has been shown to be an important source of infection with *Toxoplasma gondii* for consumers in Europe [1,2,3,4,5,6]. While consumption of undercooked meat is an infection risk factor, pork is of particular concern, as dishes such as Mett and/or Hackepeter in Germany and Poland and carpaccio of pork fillet in Italy consist of raw pork. Moreover, products such as salami, ham and bacon are cured, salted and/or smoked, which can also fail to kill the parasite. The average EU citizen eats an estimated 32.3 kg of pork annually [7], while the average Serb eats slightly more, 35.5 kg [8]. In comparison, the annual consumption of beef by EU citizens and Serbs is just about a third of that: 10.8 kg and 12 kg, respectively [8,9]. The annual per capita consumption in the EU and Serbia over several years suggests that the demand for pork is fairly level [9]. However, the demand has been projected to decline by the end of the decade in the EU due to shifting consumer preferences regarding health as well as environmental and societal concerns [7,10]. In 2021, the pork production in the EU reached a record of 23.4 million tons, yet declined by 6% in 2022 [11,12], while in Serbia, it is a mere fraction of that, at close to 300,000 tons annually [13,14]. Production, just like demand, is projected to decline further, due in part to the African swine fever (ASF), a devastating hemorrhagic disease that Europe has been battling since 2014 using a draconian “stamping out” approach mandated in 2002 [7,10,15]. As ASF knows no borders, the disease has been present in Serbia since 2019 according to the WAHIS portal [16], while “stamping out” has been mandated since 2010. In 2021, a country-wide systematic surveillance and diagnostic monitoring program for ASF was introduced [17].

Despite being lethal to domestic pigs and wild boar, ASF is not zoonotic and, thus, of no risk to public health. It is interesting to speculate, however, whether the negative impact on pig farming along with shifting consumer preferences regarding pork could, in fact, lead to a serendipitous reduction in *T. gondii* infection in the EU by the end of the decade. Whether the same can be expected in Serbia is unclear, as pork production slightly increased since the ASF outbreak [13]. Another issue is that, in Serbia, pigs are often reared and slaughtered at small farms, family farms and homesteads, some of which are registered for pork production, while others use the pork for their own consumption. Thus, the pork consumed in Serbia originates both from large farms with intensive rearing and from extensively reared “backyard pigs”. As backyard pigs usually enjoy outdoor access, which facilitates foraging for food and consuming water from environmental sources, it follows that infections with environmentally transmitted zoonotic microorganisms, such as *T. gondii*, are a public health concern. Ironically, outdoor access and foraging are hallmarks of organic farming, which is positively associated with animal welfare and consumer preferences, but also poses a risk for *T. gondii* infection [1,18,19]. Consequently, backyard pigs, just like organically reared pigs, may represent a risk to public health.

Farm biosecurity measures against *T. gondii* infections are primarily aimed at preventing oocyst ingestion. Oocysts are formed after sexual reproduction of the parasite in the gut of the definitive hosts (Felidae) [20,21,22,23]. After excretion in feces, oocysts, which can number several million, undergo sporulation, which results in the formation of infective sporozoites (8/oocyst), and importantly, in the reinforcement of the wall to provide a heightened resistance to environmental conditions [24,25]. Sporulated oocysts remain viable after freezing at −80 °C for up to two weeks, after heating at up to 70 °C for a few minutes and after treatment with harsh chemicals (10 min in 28% NH_4_OH and over 24 h in 5.25% bleach), as well as after exposure to UV at an energy density of 7500 J/m^2^, a level that is 25-fold higher than the limit that prevents biological damage [26,27,28,29,30,31]. Once sporulated oocysts are ingested (via soil, plants and/or water) by any warm-blooded species, sporozoites convert to tachyzoites and eventually to bradyzoites, the slowly proliferating life form of the parasite, which persist in tissue cysts for the lifetime of the infected host, thus making it a reservoir for meat-borne transmission [32]. Although tissue cysts are less robust than oocysts, killing all the parasites in meat by curing, salting and/or smoking may be difficult when the tissue cysts are situated deep within the muscle tissues. As the removal and/or destruction of oocysts in the environment is hardly feasible, the pigs must stay indoors, while oocysts are most effectively kept out of barns and pig pens by preventing cats from entering these and/or spaces where pig feed/fodder is stored. Rodent control, which may deter cats, is primarily aimed at avoiding meat-borne infections, as pigs are omnivores and may inadvertently consume dead rodents in feed and/or fodder.

Biosecurity measures and hygiene, along with feed provenance and quality control, have been rarely investigated on intensive farms in Serbia, while it stands to reason that most family farms and homesteads, where the greatest variability in hygienic conditions may be expected, essentially have little to no biosecurity measures in place. The major constraints associated with backyard farming are financial and spatial constraints, manpower (farm hands) and, most importantly, a limited knowledge and expertise on the elimination and/or reduction of risks for infections and other factors that cause animal mortality and/or the transmission of parasites, bacteria and viruses to other species, including people [33]. Furthermore, backyard farming, even when practiced for the commercial sale of pork and pork products, is essentially unregulated in Serbia, save for general guidelines on animal welfare and sanitary conditions on farms. Thus, we aimed to assess the historical data on the seroprevalence of *T. gondii* infection in pigs in Serbia (2003–2019), along with new data regarding *T. gondii* genotypes isolated from pigs, in the context of husbandry and specific socioeconomic aspects of backyard pig and pork production, in order to discuss the potential implications to public health. In addition, we present data on the seroprevalence in urban pet cats over the last two decades.

## 2. Materials and Methods

An analysis of the status of *T. gondii* infection in pigs in the context of the specifics of the pork industry in Serbia (pig and pork production, husbandry, economics and consumption) was performed by the re-evaluation of the published seroprevalence dating from 2003 to 2019 and of the *T. gondii* genotypes isolated from pigs thus far, with the addition of newly genotyped isolates (this study) and the seroprevalence in cats (this study).

### 2.1. Collection and Extraction of Data and Raw Data Processing

Pork-industry-related data for the period between 2006 and 2021 were collected from publicly available databases and resources, and included: the yearly pig production and mortality, pig imports/exports, the number of pigs slaughtered at abattoirs, the total slaughtered, the year-end total number of pigs, the amount of pork produced, the pork consumption per capita and the average prices of pigs (per kg of live weight) and of pork and corn [34,35]. Official data not publicly available (annual pork and pork product imports, and the pig farm registry) were kindly communicated to the authors by the Veterinary Directorate and the Chamber of Commerce and Industry of Serbia [36,37]. Data extracted from statistical yearbooks, FAO metadata and the STIPS database were transferred into spreadsheets (Excel) for the processing and calculation of descriptive statistics (average and median). Relevant data have been systematized and are shown in Supplementary Tables S1 (annual pork consumption) and S2 (pig and pork production in Serbia).

### 2.2. Genotyping of T. gondii Strains by MnPCR-RFLP

Genotyping was performed as previously described in [38] using the markers Alt.SAG2, SAG3, GRA6, BTUB, C-22, PK1, CS3, L358, C29-2 and APICO. The reaction and digestion mixtures were prepared as described in [39]. PCR was performed in a Veriti thermal cycler (Applied Biosystems, Foster City, CA, USA) and the PCR and RFLP products were separated by electrophoresis in 2.5% agarose gels stained with ethidium bromide and visualized with a BioDocAnalyze instrument (Biometra, Göttingen, Germany). RH, Me49 and NED gDNA were used as references.

### 2.3. Detection of T. gondii-Specific IgG Antibodies by Modified Agglutination Test (MAT)

Cat sera were obtained from a commercial-referral animal diagnostic laboratory that serves the greater Belgrade area. Blood was drawn at veterinary practices and clinics for various diagnostic or regulatory reasons, with the consent of the owners given on-site. All cats were adult and owned. The sample collection analyzed herein spans from 2002 to 2020. MAT was performed as originally described by Desmonts and Remington [40] and modified according to Dubey and Desmonts [41], using paraformaldehyde-fixed *T. gondii* tachyzoites. The positivity cut-off was 1:25; end-point titration was performed with all the tested sera, and positive (sera with known specific IgG antibody levels) and negative (PBS) controls were included on each plate.

Parasite propagation in mice was approved by the Ethics Council of the Ministry of Agriculture, Forestry and Water Management of the Serbia Veterinary Directorate (decision No. 323-07-02445/2014-05/1 on 19 September 2014).

## 3. Results

### 3.1. Pig and Pork Production

**Figure 1:**
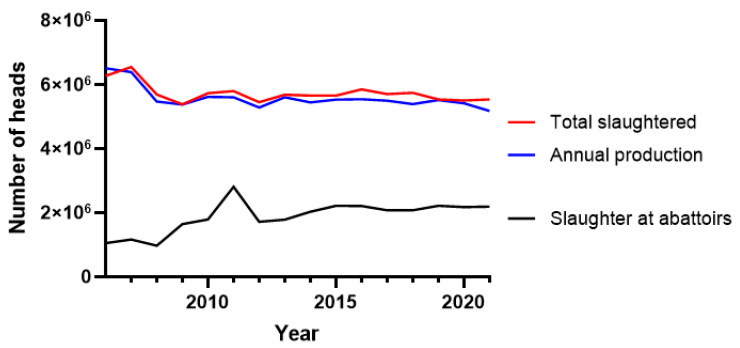
Slaughtering of pigs at abattoirs (black line) versus the slaughtered total (red line) and the annual production after accounting for mortality (blue line). The slaughtered total is an estimate based on a survey of a representative number (around 1%, differing by year) of agricultural homesteads in Serbia. In 2021, the statistical census determined that there were 560,333 agricultural homesteads in Serbia, and of these, 93.4% were family farms.

The median number of pigs in the national herd recorded at the beginning of each year from 2006 to 2021 was 3.26 million, while the median pig production was 5.8 million (Table S2). The median number of slaughtered pigs over the same period was 5,687,000, which represents nearly the entire annual production after accounting for mortality, yet only 2,055,243.5 heads (36.1%) were slaughtered at abattoirs (Figure 1). The median annual mortality was 267,000 (4.6% of the production) andshowed a decreasing trend over the years (Table S2). The median annual pork production in the last 15 years was 274,500 tons of meat. There has been a constant reduction in the number of pigs since 2007, from 4 million heads at the beginning of the year to 2.9 million at the beginning of 2021, which is a net reduction of 25.5% (Table S2).

From 2006 to 2019, 357,000 heads were exported in total (data for 2020 and 2021 are not available yet), while the sum of imported live pigs in the same period was slightly over 1.4 million heads, yielding a ratio of 3.9:1 in favor of imports (Table S2). The amount of imported meat (fresh and frozen) and meat products represents 10–20% of the domestic median annual meat production in the last 15 years [37].

**Figure 2:**
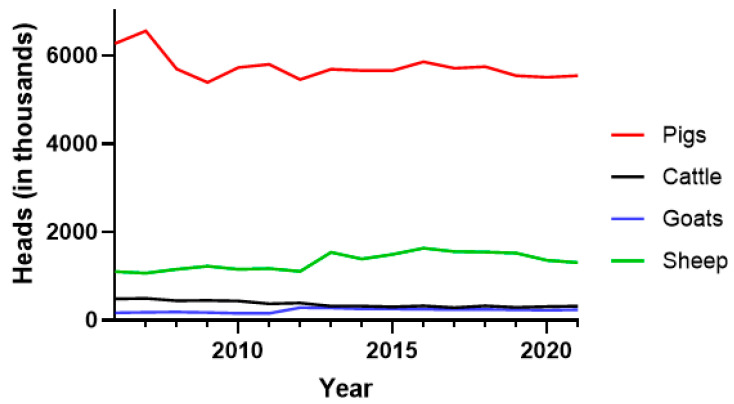
Annual slaughter of different livestock species (heads) in Serbia between 2006 and 2021. Red line—pigs; black line—cattle; blue line—goats; green line—sheep.

In terms of large livestock slaughtered from 2006 to 2022, the median number of pigs exceeded that of sheep by 4.3-fold, cattle by 17.5-fold, and goats by 23.8-fold (Figure 2).

### 3.2. Basic Economics of Pork Production

The median prices of live animals at the livestock markets and slaughterhouses, together with the price of corn in Serbia and at the trade market and the cost associated with producing one kg of pork between 2006 and 2021, are shown in Table 1. The data indicate that the most valuable commodity over the last 15 years was piglets under 15 kg, followed by piglets between 16 kg and 25 kg, while fattened pigs and sows were the least valuable, despite consuming the most corn over their lifetime. According to Serbian retail prices, the average cost of the production of a fattened pig of 120 kg (86.4 kg of meat), based just on corn and when accounting for an average weaning weight of 6.5 kg (4.68 kg of meat) [42], was an estimated USD 55.56 in 2006, while the value of a pig on the livestock market was USD 170.40. In 2011, the cost was USD 81.72 and the value was USD 208.80; in 2016, the cost was USD 70.28 and the value was USD 144; and in 2021, the cost was USD 107.87 and the value was USD 178.80. The profit, therefore, rose slightly in 2011 as compared to 2006, and then dropped sharply in 2016 and was the lowest in 2021.

### 3.3. T. gondii Infection in Pigs in Serbia (2003–2019)

The seroprevalence of *T. gondii* infection as determined by MAT in market-weight (MW) pigs slaughtered in abattoirs was 15.2% in 2003, and appears to have remained nearly the same, at 15.1%, 16 years later (Table 2). The odds ratio (OR), indicating the risk of infection for sows, vs. the MW, and for fattened vs. farrow-to-finish (FTF) pigs, was nearly the same in 2003 and 2010. Importantly, while the risk rose sharply for sows vs. MW pigs in 2019, it remained nearly the same in 2003 and 2010 for fattened vs. FTF pigs. The seroprevalence in an indigenous-breed outdoor herd (Mangulitsa) was 66.7%. Thus far, only ToxoDB#1 (13/15, with six in this study) and ToxoDB#2 (2/15) have been isolated from pig tissues (Table 2).

### 3.4. Toxoplasmosis in Owned Domestic Cats

The seroprevalence of *T. gondii* infection in owned adult cats was 33.2% (62/187). The median reciprocal titer was 400; the lowest was 25, while the highest, detected in just one cat, was 25,600.

## 4. Discussion

The official records show that, from 2006 to 2021, the lion’s share (63.9%) of the annually slaughtered pigs were not slaughtered at abattoirs, but on small farms, family farms and homesteads, indicating that much of the pig rearing and keeping happens in backyards [13]. The census of backyard pigs was kept as a separate category in statistical bulletins and yearbooks from 2006 to 2010, and the records from those years indicate that only a fifth of the national herd (~795,000 heads) did not originate from small farms, family farms and homesteads. As this category has not been available since 2010, the ratio of backyard pigs to intensively reared pigs is difficult to ascertain. Moreover, as the population size of backyard pigs is derived from self-reporting, the size of the national herd, as well as the annual pork and pig production and consumption, could be over- or underestimated. The median annual pork production from 2006 to 2021 represents an annual share of 108.9 kg per household [48]. Given the estimated per capita consumption, this annual share feeds three consumers and, thereby, statistically meets the demand of the average household of 2.6 people [48]. The popularity of pork in Serbia is attested to by the fact that more pigs are slaughtered annually than other large livestock species and, evidently, the national herd alone cannot meet the demand, as not only live pigs, but also up to a median of 36,000 tons of pork and pork products were imported annually in the last 10 years [37]. What is important from a public health perspective is the provenance of the marketed pork and pork products, as part of the national herd are backyard pigs, imported pigs are fattened on local farms and nearly all of them are destined for the pork industry. Thus, of primary concern are husbandry and local zoohygienic conditions on farms.

Over the last 14 years, a quarter (25.5%) of the national herd was lost [13]. In terms of economics, high prices associated with rearing and fattening, driven, among other factors, by fluctuations in the price of corn (the main ingredient in livestock feed and a staple food of backyard pigs) and paired with the low commercial value of live pigs, significantly reduced the profits in 2016 and made pig farming nearly unprofitable in 2021. Even the largest farm-to-fork producer in the country reduced their stock by 40,000 heads from 2016 to 2023 [49,50]. In 2016, the self-reported combined annual pig production capacity of the two largest farms was 305,000 heads, while the projected capacity for 2023 is 255,000, which is a reduction of 16.4% [49,51]. However, the motivation to keep and slaughter livestock in the backyard is deeply rooted in Serbian tradition, while the inherent commodity value of backyard pigs as food and/or bartering goods makes backyard farming very resilient, no matter the commercial profitability. It is, thus, likely that medium-sized farms, which are highly vulnerable to cost/value fluctuations, have suffered the greatest loss in market share. As a result of the declining domestic production, the import of live pigs has been steadily rising, and by 2021, it outnumbered exports by nearly 4:1.

Betić et al. [6] established that in 2019, 15 operators processed 38% (~850,000) of the pigs slaughtered at abattoirs that year. The majority (58.2%) of the suppliers of these abattoirs were large farms with a capacity exceeding 500 pigs, while 26.8% were farms with a maximum capacity of 150 [52]. From 2006 to 2023, the Serbian pig farm registry recorded 2447 entries—43.8% with a capacity of up to 150 pigs, while 20.6% exceeded 500 [36]. At least 75 entries (3.1%) were family farms and homesteads raising backyard pigs, based on their maximum reported capacity of just 20 pigs. Collectively, the data imply that the most processive abattoirs are supplied primarily by large farms that practice intensive rearing, but as medium-sized farms, farming coops and merchants are also among the suppliers, it stands to reason that backyard pigs are likely delivered to these abattoirs as well [52]. Farm registration is legally required for supplying abattoirs and the commercial sale of pork and pork products; yet, as evident from the survey report of the country-wide statistical account of agricultural homesteads, which shows that there were 560,333 in 2021 and 576,384 in 2022, the number of registered pig farms is merely a tiny fraction [13]. Moreover, 93.9% and 96.1% of agricultural homesteads in 2021 and 2022, respectively, were family farms [13]. Although pig farm registration is free-of-charge and constitutes one of the eligibility criteria for government subsidies, many subsistence and family farmers who may practice private sale of pigs, pork and/or pork products to merchants and/or coops clearly have no incentive to register. While the true share of backyard pigs in the pork industry is impossible to calculate precisely, the numbers of pigs slaughtered in and outside abattoirs suggest that they may contribute up to 60% of the pork and pork products.

The seroprevalence of *T. gondii* infection determined by MAT in MW pigs delivered to abattoirs prior to 2006 was 15.2% [44], and it was 15.1% in 2019 [6]. These data may be directly compared with the seroprevalence most recently determined by a direct agglutination test (DAT) in pigs from small and medium holdings in Poland, which was slightly lower, at 11.9% [53]. Interestingly, the seroprevalence in pigs produced on intensive farms, but finished at private farms in Cuba was 13.3% as determined by a commercial ELISA, while the seroprevalence in sows was higher (21.9%), as expected [54]. In comparison, the seroprevalence in fattened pigs reared and finished at intensive farms in Denmark, also determined by a commercial ELISA, was just 2%, while it was 19% in sows, yet it was 11% in organically reared finishers and a notable 60% in organically reared sows [19]. In Romania, the seroprevalence in backyard pigs slaughtered at family farms for the family’s own consumption was 46.8%, as assessed by IFAT [55]. While the prevalence values obtained by MAT/DAT, ELISA and IFAT cannot be directly compared, the samples were collected during the same time interval as those of Betić et al. [6], which makes them relevant. It is evident from these and similar reports that intensive pig farming is associated with the lowest, while organic farming is associated with a higher prevalence of *T. gondii* infection, respectively. This difference is due primarily to differences in biosecurity and husbandry. The nearly identical seroprevalence in MW pigs in Serbia 16 years apart is intriguing, given all the described dramatic changes that affected the pig industry during that period, and it points to the consistent presence of *T. gondii* on farms.

The true prevalence of *T. gondii* infection in backyard pigs in Serbia is not known at present, but it stands to reason that it varies considerably among farms. An indicative value may be 66.7%, as reported in Mangulitsa pigs from the Zasavica reserve [47]. Mangulitsa is an indigenous breed that is produced extensively and traditionally reared outdoors year-round. Mangulitsa pigs are usually significantly older than fattened pigs at the time of slaughter, and their meat is often cured and/or fermented into various products, which have a delicatessen status in Serbia [44,47]. The largest Mangulitsa farm is family-owned and maintains up to 1000 animals on a 70-hectare homestead in a remote rural area [56], while the Mangulitsa of the Zasavica reserve are reared on a 300-hectare pasture [47,57]. As semi-feral pigs, Mangulitsa are essentially reared without any biosecurity and hygiene measures that could prevent *T. gondii* infections. In fact, infection is facilitated, as Mangulitsa are sustained by the consumption of food and water from the environment. Indeed, *T. gondii* oocysts have been detected in rivers in Serbia [58], and their presence in soil has been demonstrated by the isolation of *T. gondii* from birds that feed off the ground, such as backyard chickens (2/8) and city pigeons (5/70) [59]. Although Mangulitsa husbandry is breed-specific, it may not significantly differ from backyard husbandry. In fact, the Mangulitsa may very well be the original backyard pig of Serbia, and just like for Mangulitsa, the main transmission mode for backyard pigs is likely to be environmental [60].

According to the available scientific data, primarily from large dairy and pig farms, “good” and “excellent” conditions on livestock farms are rare in Serbia [33,61,62]. Biosecurity was recognized as a concern over a decade ago in the recommendations of the fourth task force meeting of the classical swine fever sub-group, which mentioned the need for improvements at pig farms [63]. The report additionally estimated that in 2011, 97% of pig farms in Serbia were, in fact, backyard farms, which suggests that the number of backyard farms has been fairly constant for over a decade [13,63]. In 2022, the EU funded the “reinforcement of animal health and welfare” project, which again promoted improvements to biosecurity standards [64]. Although it is unclear how much progress has been made, a sharp drop in piglet mortality after 2012 and a subsequent leveling off suggest that major hygiene problems at farms may have been remedied. However, conditions are likely still substandard on some farms, since the average number of pigs finished per sow per year is 19, which is well below the EU range for 2018 of 25–31 [65,66].

The most numerous definitive host species of *T. gondii* in Serbia is the domestic cat. According to the results presented in this study on the presence of specific anti-*T. gondii* antibodies in cat sera collected over nearly two decades, up to a third of the cats originating from Belgrade are infected. All cats were adults with presumed little access to environmental sources of infection, as most were owned by people living in apartments. Thus, it is likely that the parasite was transmitted via the meat of infected livestock, which points to livestock as reservoirs and farms as sources of infection for livestock. Collectively, the unchanging seroprevalence in pigs and the high prevalence in cats implies that conditions on farms in terms of *T. gondii* transmission may not have changed much in over a decade. It is of concern that apparently no significant changes specifically aimed at reducing the *T. gondii* infection prevalence were made to the hygiene, husbandry or biosecurity on farms. Given the relative robustness of oocysts, hygienic measures such as disinfectant barriers (boot dips) at entrances, which may be successful in preventing infections by other microorganisms, seem less reliable for preventing oocyst introduction into the indoor holding spaces. Oocyst resistance to chemicals has been tested under different conditions, and these tests have yielded inconsistent results, which may also indicate that the efficacy of some disinfectants is underestimated [31]. Even if a disinfectant is efficacious, an additional confounding issue is that disinfectant barriers require regular maintenance (topping up and replacement). The idea that proper maintenance, rather than presence on site, is key to biosafety has been argued by Betić et al. [6], and perhaps should serve as a warning to those designing and interpreting epidemiological questionnaires. Interestingly, even if there has not been an increase in the prevalence of *T. gondii* infection in pigs for over a decade, we should still be worried about the pork and pork products from backyard pigs, but to what extent [67]?

Namely, the presence of antibodies to *T. gondii* in animals does not always correlate with the presence of parasites, and conversely, the absence of antibodies does not necessarily rule out the presence of parasites [68]. Whether this is due to biological, procedural, technical and/or analytical factors is a matter of debate, but in pigs, up to 5% of seronegative animals could harbor parasites [68]. In fact, according to Kuruca et al. [47], one seronegative pig yielded a positive mouse bioassay, as confirmed by an observation of tissue cysts (ToxoDB#1) in the brain tissue. Similarly, ToxoDB#54 was isolated from a seronegative horse [69]. So far, 13 strains (out of a total of 15) isolated from both extensively and intensively reared pigs were ToxoDB#1, which includes all the strains (*n* = 6) genotyped in this study, while the remaining two strains from a previous study were ToxoDB#2 [46]. All of the six isolates genotyped herein were from sows sold to abattoirs by merchants. As intensive farms usually deliver their own pigs to abattoirs, it is likely that these sows were purchased at livestock markets and/or small farms. We have previously reported that all these sows were seropositive, with MAT titers ranging from 1:50 to 1:400, while 4/6 had a titer of 1:100 [6]. The genotyping results are entirely expected given the population structure of *T. gondii* in Serbia [39] and Europe [70]. The results are also consistent with other published studies; namely, in Romania, ToxoDB#1 was isolated from backyard pigs [55], while in Spain, ToxoDB#1 and ToxoDB#2 were isolated from free-ranging Iberian pigs [71].

However, as genotypes other than archetypes also circulate in Serbia, albeit at a lower frequency [39], future isolates from pigs may not all be archetypes. As long as the imported pigs and pork originate from Europe, the risk of the introduction of genotypes of non-endemic lineages is low. However, this may change if the national herd continues to decrease and if ASF is not “stamped out” entirely in the EU in the coming years, since pigs and pork may need to be sourced from outside of Europe. This may lead to a perturbance in the *T. gondii* population structure, which may ultimately affect host species biodiversity and ecosystem balance. Therefore, from a *T. gondii* population genetics point of view, it is perhaps not the presence of backyard pigs in our national herd one should worry about, but rather their disappearance. However, the prevalence of infection in pigs and the pork consumption data indicate that there is a significant potential to acquire *T. gondii* infection from the pork of backyard pigs. In addition, as presented here, the economics of pork production favors backyard farming, while the prevalence in pet cats also suggests that farms are a *T. gondii* reservoir. Thus, there is a clear public health risk associated with backyard farming that should not be ignored.

## 5. Conclusions

The implementation of specific biosecurity measures at family farms and homesteads for the commercial sale of their own pork and pork products or their own consumption is currently not mandated in Serbia by law. The most effective biosecurity measure proven to prevent *T. gondii* infection, controlled indoor rearing, is inherently inconsistent with backyard farming. Thus, the development of innovative, cost-effective and implementable measures suitable for backyard farming and research into their efficacy should be a priority. In the meantime, educating farmers regarding risk factors for *T. gondii* infection in livestock, and importantly, providing guidelines and advice on how to prepare pork and pork products that are safe for consumption, may be the most appropriate preventive measures in countries that practice backyard farming.

## Figures and Tables

**Table 1:** Average prices in USD per kg of live weight at livestock markets ^1^ and slaughterhouses ^2^ for pork and average prices in USD for corn (Serbian and trade market prices) in 2006, 2011, 2016 and 2021. Weekly currency conversion factors were taken from the Serbian agriculture market information system database and averaged for the year. The price of 1 kg of corn in Serbia was calculated based on the retail cost of a 50 kg bag. The price of corn at the trade market was adjusted from a bushel of corn (25.4 kg) to 1 kg. For the cost of corn per kg of pork, the US Grains Council conversion factor of 4.55 kg was used [43].

Year	Sows	Piglets (<15 kg)	Piglets (16–25 kg)	Fattened Pigs (80–120 kg)	Fattened Pigs (>120 kg)	Price of Corn in Serbia	Trade Market Price of Corn	Cost of Corn/kg of Pork	Pork Retail
2006	1.18 ^1^	2.13 ^1^	1.89 ^2^; 1.99 ^1^	1.42 ^1^	1.26 ^1^	0.15	0.15	0.68 ^3,4^	5.24
2011	1.35 ^1^; 1.48 ^2^	2.03 ^2^; 2.16 ^1^	2.03 ^2^; 2.16 ^1^	1.74 ^1^; 1.76 ^2^	1.48 ^2^; 1.62 ^1^	0.22	0.27	1.00 ^3^ 1.22 ^4^	5.78
2016	0.94 ^1^; 0.91 ^2^	1.77 ^1^; 1.6 ^2^	1.6 ^1,2^	1.20 ^1^; 1.21 ^2^	1.06 ^1,2^	0.19	0.14	0.86 ^3^ 0.66 ^4^	3.84
2021	1.2 ^1,2^	2.24 ^2^; 2.39 ^1^	2.29 ^1,2^	1.49 ^1,2^	1.4 ^1^; 1.32 ^2^	0.29	0.22	1.32 ^3^ 1.02 ^4^	5.29

^3^ Based on price in Serbia. ^4^ Based on world trade market price.

**Table 2:** *Toxoplasma gondii* infection in pigs in Serbia: seroprevalence, selected infection risk factors (age and production type) and genotypes.

Number of Pigs	Sample Origin (Year of Sampling)	Seroprevalence (%)	Adjusted OR	Genotype	Reference
605	Farms; abattoirs (2003)	28.9 (15.2 ^2^)	Sows vs. MW: 3.87 Fattened vs. FTF: 3.96	- ^4^	[44]
488	Abattoirs ^1^ (2010)	9.2 (8.3 ^2^)	Sows vs. MW: 4.71 Fattened vs. FTF: 3.95	-	[2]
182	Abattoirs (2015)	17 ^2^	-	ToxoDB#1 ToxoDB#2	[45,46]
825	Abattoirs (2019)	16.5 (15.1 ^2^)	Sows vs. MW: 14.25 Fattened vs. FTF: 4.51	ToxoDB#1	[6]; This study
18	Private slaughterhouse (2015)	66.7 ^3^	-	ToxoDB#1	[46,47]

^1^ Belgrade region only. ^2^ Market-weight only (MW). ^3^ Outdoor herd. ^4^ Not available.

## Data Availability

Most of the data presented herein are publicly available from the cited references and supplementary material. Exceptions include the Serbian national listing of registered homesteads/farms/intensive-production pig farms, and a listing of pig imports, which was provided by the Serbian veterinary authorities upon special request from the authors.

## Supplementary Materials

The following supporting information can be downloaded at: https://www.mdpi.com/article/10.3390/microorganisms11071857/s1, Table S1: Worldwide pig meat consumption, 2014–2018; Table S2: Pig (in heads) and pork (in tons) statistics from 2006 to 2021 in Serbia.
