# Supplementary material for: A Pork Industry in the Backyard: An Analysis of Toxoplasma gondii Infection in Serbia’s Pigs
Source: Microorganisms. 2023 Jul 23;11(7):1857. doi: 10.3390/microorganisms11071857 (PMC10385063; doi:10.3390/microorganisms11071857)
Supplement: Supplementary file 1 [file microorganisms-11-01857-s001.zip › Table S2.pdf]

Supplementary material - Table S2

A Pork Industry in the Backyard: An Analysis of *Toxoplasma gondii* Infection in Serbia's Pigs

Aleksandra Uzelac [1,†], Nikola Betić [2,†], Nedjeljko Karabasil [3], Vladimir Ćirković [1], Olgica Djurković-Djaković [1] and Ivana Klun [1,*]

[1] Centre of Excellence for Food- and Vector-borne Zoonoses, Institute for Medical Research, University of Belgrade, 11129 Belgrade, Serbia

[2] Institute of Meat Hygiene and Technology, 11000 Belgrade, Serbia

[3] Department of Food Hygiene and Technology, Faculty of Veterinary Medicine, University of Belgrade, 11000, Belgrade, Serbia

* Correspondence: iklun@imi.bg.ac.rs; Tel.: +381-11-2685-788

Table S2. Pig (n of heads) and pork (in tons) statistics from 2006 to 2021 in Serbia. The number of animals in the national herd is in bold type. Source: Statistical Office of the Republic of Serbia. Statistics of Agriculture, forestry and fishery [13].

Year	Starting total	Production	Mortality	Import	Export	Slaughter at abattoirs	Total slaughtered	Year-end total	Pork produced[1]
2006	**3,759,000**	7,360,000	852,000	0	1,000	1,052,765	6,267,000	**3,211,597**	255,000
2007	**4,000,000**	7,195,000	805,000	3,000	8,000	1,161,255	6,553,000	**3,831,894**	289,000
2008	**3,832,000**	6,081,000	612,000	3,000	21,000	970,267	5,690,000	**3,594,236**	266,000
2009	**3,594,000**	5,968,000	593,000	71,000	25,000	1,642,735	5,385,000	**3,631,013**	252,000
2010	**3,631,000**	6,237,000	619,000	10,000	42,000	1,791,091	5,728,000	**3,488,738**	269,000
2011	**3,489,000**	6,162,000	556,000	23,000	36,000	2,807,225	5,795,000	**3,286,900**	271,000
2012	**3,287,000**	5,805,000	524,000	55,000	32,000	1,714,006	5,453,000	**3,138,508**	258,000
2013	**3,139,000**	5,822,000	219,000	118,000	31,000	1,782,565	5,684,000	**3,144,207**	249,000
2014	**3,144,000**	5,668,000	223,000	330,000	26,000	2,031,428	5,657,000	**3,235,658**	258,000
2015	**3,236,000**	5,763,000	232,000	217,000	46,000	2,217,744	5,654,000	**3,284,378**	278,000
2016	**3,284,000**	5,824,000	283,000	77,000	28,000	2,211,838	5,853,000	**3,021,167**	301,000
2017	**3,027,000**	5,725,000	229,000	123,000	23,000	2,079,059	5,706,000	**2,910,525**	307,000
2018	**2,911,000**	5,611,000	224,000	252,000	22,000	2,079,059	5,745,000	**2,910,525**	303,000
2019	**2,782,000**	5,733,000	218,000	160,000	16,000	2,218,968	5,538,000	**2,903,007**	298,000
2020	**2,903,000**	5,670,000	251,000	163,000	na	2,176,449	5,502,000	**2,983,102**	299,000
2021	**2,983,000**	5,415,000	240,000	246,000	na	2,189,719	5,536,000	**2,868,121**	307,000

[1] Tons
